# Growth and establishment of monodominant stands affected by ENSO and flooding in the Pantanal

**DOI:** 10.1038/s41598-020-60402-x

**Published:** 2020-02-25

**Authors:** Darlene Gris, Eliana Paixão, Rosani C. O. Arruda, Iria H. Ishii, Maria R. Marques, Geraldo A. Damasceno-Junior

**Affiliations:** 1 Programa de Pós-Graduação em Ecologia e Conservação, Universidade Federal de Mato Grosso do Sul (UFMS), Campo Grande, Mato Grosso do Sul 79070-900 Brazil; 2 Grupo de Pesquisa em Ecologia Florestal, Instituto de Desenvolvimento Sustentável Mamirauá (IDSM), Tefé, Amazonas Brazil; 3 Programa de Pós-Graduação em Ecologia e Conservação da Biodiversidade, Universidade Federal de Mato Grosso (UFMT), Cuiabá, Mato Grosso 78060-755 Brazil; 4 Laboratório de Anatomia Vegetal, Instituto de Biociências, Universidade Federal de Mato Grosso do Sul (UFMS), Campo Grande, Mato Grosso do Sul 79070-900 Brazil; 5 Laboratório de Botânica, Campus do Pantanal, Universidade Federal do Mato Grosso do Sul (UFMS), Corumbá, Mato Grosso do Sul 79304-020 Brazil; 6 Laboratório de Bioquímica, Instituto de Biociências, Universidade Federal de Mato Grosso do Sul (UFMS), Campo Grande, Mato Grosso do Sul 79070-900 Brazil; 7 Laboratório de Ecologia Vegetal/Laboratório de Botânica, Instituto de Biociências, Universidade Federal de Mato Grosso do Sul (UFMS), Campo Grande, Mato Grosso do Sul 79070-900 Brazil

**Keywords:** Climate-change ecology, Population dynamics, Wetlands ecology, Plant ecology, Climate-change ecology

## Abstract

Climatic factors can influence the establishment and growth of wood species, but little is known about the effect of these factors on monodominant communities in wetlands. Therefore, we asked how climatic factors, such as ENSO (*El Niño*-Southern Oscillation), precipitation and flooding, influence growth and establishment of the dominant species *Erythrina fusca* in the Pantanal. We determined the age of sampled individuals, the age of the population and evaluated the effects of climate on tree growth. We obtained samples for dendrochronological analyses using destructive (seven individuals) and non-destructive methods. We cross-dated and built a chronology, correlating results with climatic factors. We sampled 0.6 ha of the population and separated individuals into diameter classes to determine age based on diameter/age ratio obtained through dendrochronological analyses. We obtained a chronology with individuals up to 34 years old, while in the population sample, the oldest individual was 54 years old. The factors that influenced growth during the study period were precipitation (positive correlation) and *El Niño* (negative correlation). *E. fusca* individuals seem to grow more during the period of highest precipitation, and *El Niño* events reduce precipitation in the Pantanal, resulting in a decrease in the growth of *E. fusca* individuals. We detected a decrease of young individuals in the last nine years, which seems to be related to the decrease in minimum flood levels. This indicates a future decline in the number of individuals. These results allow us to propose measures to protect these monodominant formations, which mainly involve avoiding further anthropic activities, that could reduce flooding levels.

## Introduction

In tropical areas, some tree species can occur as monodominant stands in which more than half the total number of arboreal individuals belong to only one species^1,2^. Many features and events can influence monodominance, the most common being flood, fire, succession, fungal conditions, and edaphic factors^3–6^. These factors act as selective pressures that exclude less tolerant species, allowing more tolerant species to dominate the environment^3,4^.

Knowledge about the factors that affect the dominance of a species can be gained through an understanding of its dynamics and structure. The study of tree-rings is one way to interpret both the dynamics of vegetation and estimate the growth rates of arboreal species^7^. The data provide clues to further understand how climate change can affect vegetation in the future, thereby arming researchers with the knowledge needed to formulate strategies to conserve these formations^8^. In tropical regions, the monodominance of a species is often associated with seasonal flooding^3,9^. Some studies show that flood pulse and precipitation directly influence the growth and establishment of species^10–12^. When we associate climatic variables with wood increment patterns in some species, we can begin to understand how climate and natural events affect tree growth^13,14^ and, perhaps, the establishment and growth of monodominant stands. One such natural event is *El Niño* (*El Niño* Southern Oscillation - ENSO), which, in the Pantanal, causes a significant reduction in precipitation, thus affecting the growth of different species, such as *Vochysia divergens* Pohl^15,16^.

Environmental factors can also influence the establishment and development of individuals of different tree species, e.g. the spatial arrangement and the interactions between their parent plants and the past environmental characteristics, all affect the spatial structure and distribution of populations^17^. Therefore, the age structure of a current population reflects the conditions of recruitment and the mortality rates to which the populations were subjected^17^. In this context, the Pantanal is a favorable environment to study the occurrence and factors that influence species dominance, in particular because many monodominant species are found in its floodplains, such as *Tabebuia aurea* (Silva Manso) Benth. ex S. Morre (popularly known as “paratudal”), *Byrsonima cydoniifolia* A. Juss. (“canjiqueiral”), and *Copernicia alba* Morong ex Morong and Britton (“carandazal”), or *Erythrina fusca* Lour. (“abobral”), among others^18^.

The monodominant stands of *E. fusca* which occur in the Pantanal in the subregion of Cáceres, are an important resource in this subregion. *E. fusca* is a pioneer species, heliophyte and tolerant to flooding^19,20^. Its flowers are fleshy, showy, and frequently visited by birds and pollinators. The dry legume fruit produces numerous seeds, which, during dehiscence, fall into the river and are normally ingested by fish^21,22^. Furthermore, the roots present a particular arrangement that provides shelter for a variety of species, especially reptiles and mammals. Therefore, studies that report on the establishment and growth of a monodominant species in relation to environmental features are important for maintenance and conservation of these stands.

Usually, growth is negatively affected by inundation; therefore, the growth of woody species occurs principally during the dry season, when flooding and precipitation levels are lower, and stagnates during the wet season, when the flooding and precipitation levels are higher^12,23,24^. Our hypothesis is that the establishment and growth of individuals of *E. fusca* in the monodominant stands in the Pantanal subregion of Cáceres is negatively influenced by higher flooding and higher precipitation levels and positively influenced by the occurrence of *El Niño* since this event can cause significant precipitation reduction. Therefore, in this study, we aimed to build a chronology of *E. fusca* growth from monodominant stands in the Pantanal sub-region of Cáceres, verify how the structure of the monodominant population is related to variations in inundation and climate, and evaluate effects of climate (local and global) and inundation on its growth. We intended to obtain data to support the conservation of these monodominant stands, considering their ecological importance in the study region.

## Methods

### Study area

The Pantanal is a vast floodplain crossed by the Paraguay River and its tributaries, extending throughout Brazil, Paraguay, and Bolivia, with an area of approximately 140,000 km^2^^ 25^. This large wetland has a predictable and monomodal flood pulse, with low amplitude and long duration^26^, and the structure and dynamics of the Pantanal are dependent on the fluctuating levels of flooding^10,27^.

**Figure 1:**
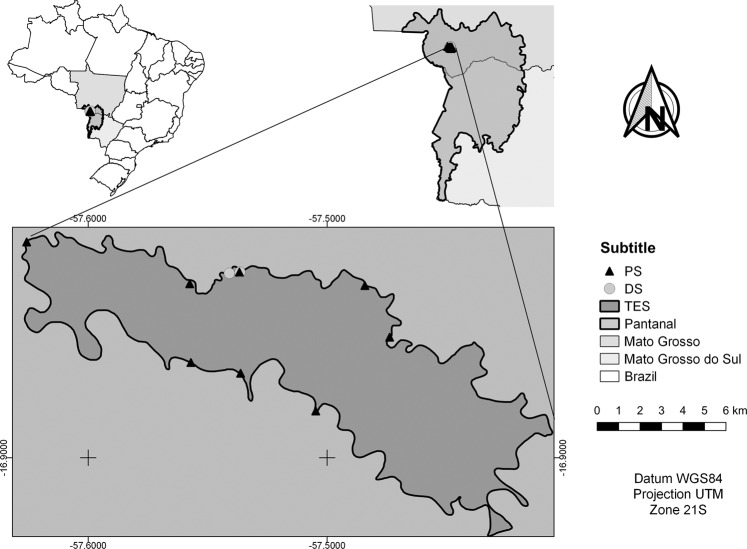
Monodominant stands of *E. fusca* sampled from population structure analysis (PS) and areas of destructive sample collection (DS) at Taiamã Ecological Station (TES) in the northern Pantanal subregion of Cáceres, Mato Grosso State, Brazil.

We conducted the study at Taiamã Ecological Station (TES) (Fig. 1) and the nearby areas of Sararé Island and the Jubran Private Reserve in the Pantanal subregion of Cáceres where it is possible to find floodplains covered by grasses and other herbaceous plants, floating meadows (*batumes*), pioneer forests, and large monodominant stands of *E. fusca*, locally known as *abobral*^28^. We created the map using the software QGis Versão 3.4^29^, all the shapefiles used are open access. The shapefiles of Brazilian states was provided by the Instituto Brasileiro de Geografia e Estatística^30^, Pantanal provided by Ministério do Meio Ambiente^31^ and TES provided by Instituto Chico Mendes de Conservação da Biodiversidade^32^.

**Figure 2:**
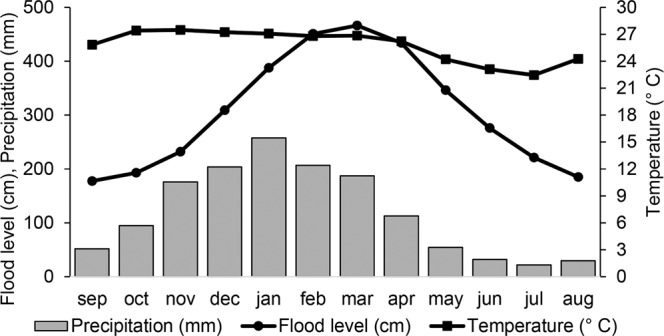
Average flood levels of the Paraguay River, annual precipitation, and temperature between 1980 and 2014 in the northern Pantanal subregion of Cáceres, Mato Grosso, Brazil.

The climate is seasonal Aw in the Köppen classification^33^ with a dry season from May to September and a rainy season from October to April^15^. In the Pantanal subregion of Cáceres, high flooding levels almost coincide with the rainy seasons (Fig. 2). The average annual temperatures are around 26 °C, ranging from a minimum average of 20 °C to the maximum of 31 °C, and the average annual precipitation is 1227 mm. We calculated these values for the years between 1980 and 2014 from data obtained from the Instituto Nacional de Meteorologia (Brazilian National Institute of Meteorology)^34^ and the Marinha Brasileira (Brazilian Navy)^35^.

In the subregion, the predominant soil is Eutrophic Gleysol^36^, which remains periodically saturated by water stagnation or the occurrence of lateral flow in the soil. The striking feature of this soil type is its strong gleization owing to a reduced environment wherein the water saturation throughout the year, combined with oxygen demand for biological activities, leads to low dissolved oxygen concentration in soil^37^.

Considering that the Pantanal is in Southern Hemisphere, it is important to remember that, for some species the growth of tree rings begins at the beginning of the rainy season^15,38,39^, which corresponds to October, that is, the growing year of the plant does not follow the calendar. Thus, in results (Figures) the months designated with (−1) indicate the growth season in the previous year and (+1) indicate the change of the calendar year during the growing season in the Southern Hemisphere, similar methodology is used by other authors in recent papers (e.g. Fortes *et al*.^15^; Locosselli *et al*.^40^; Locosselli *et al*.^41^).

### Species description

*E. fusca* (Fabaceae) is a tree species (20–30 meters high) with a globose treetop and short and branched trunk, prickly when young. The leaves are alternate, composite, trifoliolate, with leathery leaflets, glabrous. The inflorescence is in the form of terminal racemes, with showy yellow-orange flowers^19^. This is a pioneer, heliophyte, deciduous species, tolerant to flooding^19,20^, which occurs naturally in riparian forests and blooms from May to September, with intense fructification starting in November^19,21^. During fieldwork, we observed that adult individuals of *E. fusca* showed a pattern of prop roots which seems to help improve root oxygen levels and root fixation on the soil during flooding, we also observed that the higher the flood level, the more prop roots could be found in individuals.

Monodominant stands of *E. fusca* occur on the plains of the northern Paraguay River, in the Cáceres subregion, and along the banks of the Aquidauana River^18^. In the dry season, this species occupies an area of approximately 18 km^2^, which represents 16% of the station area^28^. These monodominant stands are low-density vegetation with predominance of arboreal individuals of *E. fusca* (approximately 77% of the community), along with another 10 tree/shrub species (Unpublished data). The herbaceous stratum is very homogeneous, dominated by grasses, and the soil is covered by a histosol layer with leaf litter, mostly composed of *E. fusca* leaves.

We collected and prepared a fertile sample of *E. fusca* according to herbarium techniques^42,43^ and deposited the voucher in the CGMS Herbarium under registration CGMS 40967.

### Collection and sample preparation

Considering that the area was a Brazilian Federal Conservation Unit (Taiamã Ecological Station) with a limited permission to cut trees (for this project we were allowed to collect only seven individuals), in December 2013, we selected two sample areas (Fig. 1) where we marked seven *E. fusca* trees of different diameters. In each tree, we used the Mariaux Windows method^44^, which starts by using a mechanical incision to remove part of the bark from the tree to expose the vascular cambium. After two years, in December 2015, we collected these trees using the destructive method. These destructive samples were included in the total sampling, but they were also used for knowledge of wood anatomy, growth ring pattern determination, and presence/absence of false or missing rings.

After previous analysis of the discs (destructive samples) and verification that there were no missing or false rings that could lead to misinterpretation of data, we decided to complement sampling with the use of non-destructive method. The non-destructive method has been used by several authors (e.g. Schöngart *et al*.^45^; Fonseca-Junior *et al*.^46^; Locosselli *et al*.^41^; Andrade *et al*.^47^; Neves *et al*.^48^) in recent research in tropical trees with good results of ring analysis. In December 2016, using the non-destructive method, we extracted cores from the stems of 29 trees (one or two cores per stem, but we used the one that allowed the best view of the rings) using an increment borer (Pressler borer) 5 mm in diameter. These non-destructive samples were collected over the *E. fusca* monodominance areas at Taiamã Ecological Station, close to where we collect the destructive samples, according to the availability of collect individuals of different diameters. In the field, we measured the diameter at breast height (DBH) with a diametric tape and the height of all trees using a hypsometer.

We kept samples at room temperature until completely dried to avoid cracking. Then we polished the samples with sandpaper of different grains (80, 100, 180, 220, 320, 400, 600, and 1200) so that the growth rings and anatomical characteristics of the wood could be visualized^24^.

### Anatomical procedures

The growth rings were delimitated with anatomical analyses. We analyzed the anatomical samples at the Laboratório de Anatomia Vegetal of Universidade Federal de Mato Grosso do Sul (UFMS). For anatomical description and determination of tree-rings, we used 1 × 1 × 2 cm wood samples of three *E. fusca* individuals. We boiled the wood samples in glycerin solution (50%) for 15 minutes to soften the wood. Afterwards, we prepared sections (longitudinal and cross planes) of about 25 µm thickness using a sledge microtome (Leica SM2000R). The sections were bleached with sodium hypochlorite, washed in distilled water and acetic acid (1%), and stained with alcian blue and safranin, both in the concentration of 1% in water, proportion of 9:1^49^. Macerations were prepared using wood segments dissociated in hydrogen peroxide and glacial acetic acid (1:1) and heated in an oven at 60 °C for 12 h. Isolated cells were stained with 1% Safranin or 12:25% basic fuchsin, in 50% ethanol and mounted in 50% glycerin solution. To detect lignified secondary wall, we performed a histochemical test with acidic phloroglucin (phoroglucinol, HCl and ethyl alcohol) in which the lignified cell wall becomes violet-red^50^. We analyzed and photographed the sample under a microscope. The descriptions of wood constituents followed the guidelines of the IAWA Committee^51^.

After defining the anatomical and growth ring characteristics, we measured the width of the tree-rings. To do this, we used a tree-ring measurement (Lintab^TM^ 6) with precision of 0.01 mm, together with TSAP-WIN^TM^ Scientific software (Time Series Analysis and Presentation), which is specific for time sequence analysis. We analyzed the samples at the Laboratório de Ecologia Vegetal of Universidade Federal de Mato Grosso do Sul (UFMS). The tree-rings were delimited and measured according to the classification of Coster^52,53^, as adapted by Worbes^24^.

### Growth modeling and population structure

After we measured the width of the rings, we generated the individual radial increase rates, from which we obtained the rates of mean diameter increment (MDI) in millimeters. We built the cumulative diameter growth curves and then fitted to the DBH obtained in the field^14^. Through adjustments of the cumulative diameter growth curves, we obtained the relationship between age and diameter of individuals^54–56^. Based on the individual cumulative diameter growth curves, we calculated the mean diameter growth curve, which was fitted to a sigmoidal regression model, to obtain the relationship between age and diameter^55,56^, according to:

$$DBH=(\frac{{\beta }_{0}}{1+{(\frac{{\beta }_{1}}{age})}^{{\beta }_{2}}})$$

where DBH is the diameter at breast height (cm) and β0, β1 e β2 are equation parameters para for the model obtained by the non-linear regression fit.

The relationship between DBH and the height of trees sampled (H) was adapted to a non-linear regression, according to Schöngart^55,56^. The regression models were produced using the Xact software (SciLab).

We sampled monodominant stands of *E. fusca* (MSEF) in eight areas (Fig. 1), distributed throughout the TES (over 115 km2), and in each area, we established three 50 × 5 m plots with a minimum distance of 20 m between them, totaling 0.6 ha. We allocated the plots in order to best show different elevations and respective flooding durations of the Paraguay River levees. Trees with circumference at breast height (CBH) of ≥15 cm were included in the sampling. Bifurcated stems were only included if at least one had a circumference ≥15 cm. We did not find individuals below 15 cm of CBH. We transformed the data of CBH into DBH (diameter at breast height) before performing analyses. We also measured the height of trees with a hypsometer.

Using population sampling data, we constructed a new correlation of diameter with height of the individuals, adapted to a nonlinear regression^55^. In addition, using the values of DBH and the age of each sample, we calculated the age of the individuals included in the population sample.

We also distributed the individuals in classes of diameter with intervals of classes defined by Spiegel’s formula^57^ constituted by A/K, where A represents the breath of data (diameter), and K represents the algorithm of Sturges: K = 1 + 3.3 log N, where N is the number of individuals sampled. Considering the relationship between diameter/age of individuals sampled for dendrochronological analyses, we calculated the age of all the individuals sampled from the population.

Since the number of individuals in the largest diameter classes tends to experience a natural decrease, we were unable to perform a direct analysis between the environmental factors and the number of individuals in each diameter class. Therefore, we used the distribution of individuals in the first five diameter/age classes (classes with more than five individuals, totaling the last 35 years), to build a series that contains maximum and minimum values of flooding levels and total precipitation data for the years falling into each class. To verify differences in the levels of these factors between age groups, we performed analysis of variance, followed by the Tukey 5% test using R software and the agricolae package^58^.

We determined the duration of flooding of each plot. First, we measured from the ground the height of the water mark left by the last inundation on tree trunks inside the plots. Then, we obtained an average of the water marks per plot and compared these values with the highest level recorded at the hydrometric gauge of Cáceres during that year of sampling. Considering that the overall topography is very flat and that no tributaries flow between the gauge and the sampled area, we considered variation in water level to be the same. Using these data, we calculated the topographic position of each plot in relation to zero by the Cáceres gauge. Thus, we used ten years of data to calculate an average period of flooding for each plot.

With the calculations above mentioned we observed that the monodominant stands of *E. fusca* in the Pantanal subregion of Cáceres were subjected to different periods of flooding, ranging from 42 to 117 days per year. Therefore, to observe the differences in the distribution of individuals in diameter and age class subjected to different flood periods, we divided the plots into two parts (i.e., half the total length of the inundation gradient) based on the results obtained by Damasceno-Junior *et al*.^59^ and Arruda *et al*.^60^. Therefore, half of the plots were in the first category (flooding between 42–78 days per year) with 62 individuals, while the second category (flooding between 79–117 days per year) presented 91 individuals. To compare the number of individuals within diameter classes between flood periods (42–78 and 79–117), we used a chi-square test with R software^61^.

### Environmental variables

We used the weather data corresponding to the period from 1981 to 2014. We obtained the precipitation data from KNMI Climate Explorer^62^. We obtained the data on daily flood level of the Paraguay River in the study subregion from the Marinha do Brasil (Brazilian Navy)^35^. The records of ENSO events *(El Niño* 1 + 2*, El Niño 3, El Niño 3.4* and *El Niño 4)*, Pacific Decadal Oscillation (PDO), and Southern Oscillation Index (SOI) were obtained from the database available on the National Oceanic Atmospheric Administration (NOAA)^63^.

### Chronology and correlations with climatic variables

We used standard dendrochronological techniques to cross-date the time series of different individuals and combine these time series with a main chronology^64^. The cross-dating process comprises overlapping individual indexed curves, matches the variations in rings width between trees and checking relationships between dating to infer where rings are missing, false or incorrectly observed^13,23,45,65,66^. We visually and statistically checked the increment curves pairwise with cross-dating to build an average chronology based on the growth ring series of all individuals^65^. We used the TSAP-WIN^TM^ software to describe the similarity between individual curves. Percentage of parallel run was used to indicate the year-to-year agreement in the oscillation of two curves within the overlapping interval^67^. The samples without the pith were carefully analyzed, paired (visually and statistically) and cross-dating with the other curve samples according to the similarity between the individual curves, the comparison of diameters and according to the pith distance, thus, it was possible to estimate the average number of missing rings, similar methodology is used by other authors (e.g. Brienen *et al*.^68^). Cross-dating was visually and statistically validated by the coefficient of coincidence Gleichlaeufigkeit (GLK) and the T value^65,69^. Both concepts are used to verify the quality of agreement between temporal series, and GLK is specially developed for growth ring cross-dating^65^. After this process, the best individual time series were selected for the construction of the average time series (chronology).

The curves standardization removes the effects of ring-width reduction associated with environmental fluctuations, changes in age and position within the stem, and site productivity conditions^66^. To remove long-term growth trends related to increasing tree age and size, or even from changes in the surrounding forest, causing effects on competitive structure^66^, a five-year moving average was used to convert crude tree-ring curves into growth indices. This procedure results in normally distributed data, which is an elementary condition for correlation with climate variables^70^.

To verify if the species is sensitive to environmental variations we calculated the mean sensitivity, which expresses the relative year-to-year variation in ring width^66^. We tested the significance of the relationships between climatic factors and chronology of *E. fusca* using correlation matrices, and we built the graphics using R software^61^.

## Results

### Anatomical description and determination of growth rings

The wood of *E. fusca* is whitish and poorly lignified. The pores are distinct to the naked eye and form semi-porous rings, with a row of larger pores at the beginning of the growth layer. These pores are sparsely distributed and small-sized toward the end of the layer. The pores are in dendritic pattern with solitary (most) and multiple clusters. The axial parenchyma is visible to the naked eye and is confluent and in bands.

**Figure 3:**
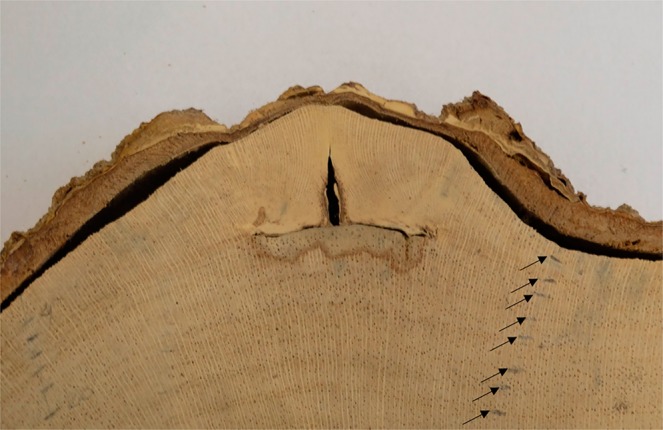
Macroscopic detail of *E. fusca* stem disk with Mariaux window scar collected in the Pantanal subregion of Cáceres, Mato Grosso, Brazil. The arrows indicate the delimitations of distinct growth layers, with a band of parenchymatic flat cells, above which we see the rest of the layer showing a decrease in pore size and number. The end of the layer is marked by few, or no pores, and a band of parenchyma with flattened cells.

**Figure 4:**
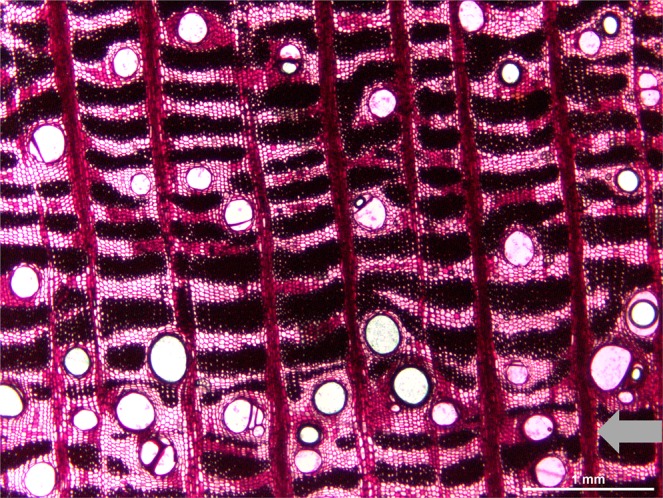
Microscopic detail of *E. fusca* wood collected in the Pantanal subregion of Cáceres, Mato Grosso, Brazil. The gray arrow indicates the region where the growth layer begins, with a higher concentration of parenchyma and large pores.

The growth layers are distinct (Fig. 3), and the beginning is marked by the distribution of the semi-porous rings and the presence of parenchyma and few fibers (Fig. 4). The rest of the layer shows a decrease in pore size and number, and the end of the layer is marked by few, or no, pores and a band of parenchyma with flattened cells (Fig. 4). When looking at the samples with Mariaux window marks, we noticed that new rings started soon after we made the scars. This means that the beginning of the growth of a new layer occurred between the months of December and January. From anatomical analysis, we observed no false, or missing, rings, allowing us to confidently perform measurements on non-destructively obtained samples.

### Growth modelling and population structure

**Figure 5:**
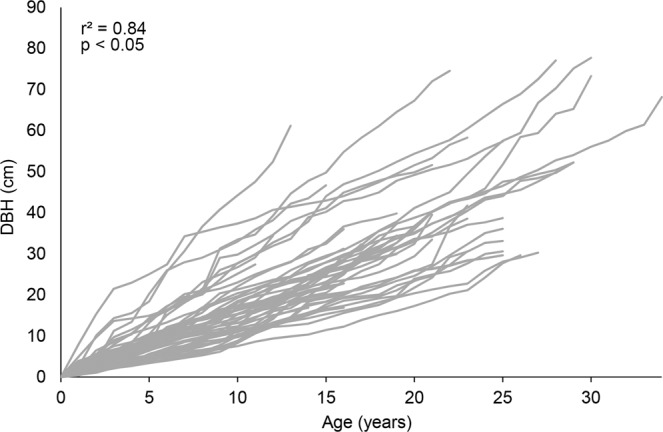
Relationship between diameter growth at breast height (DBH) and age of individuals of *E. fusca* in the Pantanal subregion of Cáceres, Mato Grosso, Brazil. Lines represent the individual growth in diameter of each sample.

The ages of sampled individuals of *E. fusca* ranged from 6 to 34 years. The relationship between age and DBH of the trees was highly significant (r^2^ = 0.84, P < 0.05, Fig. 5). The maximum diameter increment rate of *E. fusca* was observed at 7 years (1.6 cm year^−1^); after this period, the rate decreased linearly.

**Figure 6:**
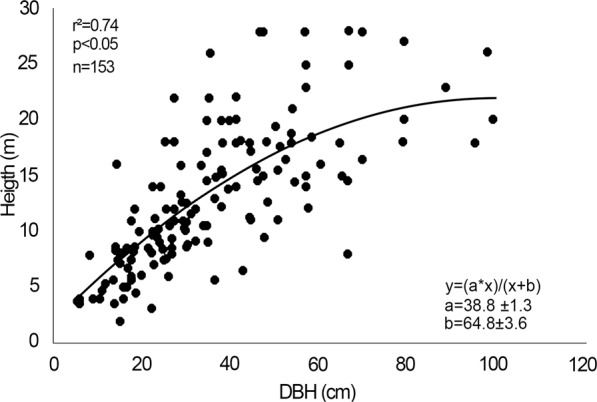
Relationship between diameter at breast height (DBH) and height of 153 individuals of *E. fusca* in the Pantanal subregion of Cáceres, Mato Grosso, Brazil, adjusted with a nonlinear regression analysis.

Diameter was highly correlated (r^2^ = 0.93, P < 0.05) with height of individuals. The same was observed for individuals in the population structure samples (r^2^ = 0.74, P < 0.05, Fig. 6), as described by a nonlinear regression model.

**Figure 7:**
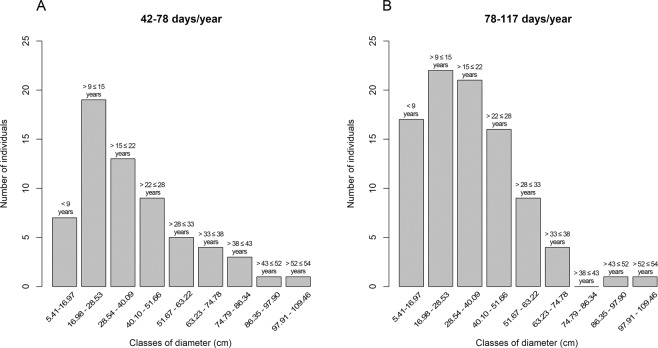
Number of *E. fusca* individuals sampled in the Pantanal subregion of Cáceres, Mato Grosso, Brazil, distributed into diameter classes (DBH) and age among classes, a) individuals occurring in areas where the flood period varies between 42–78 days/years, b) individuals occurring in areas where the flood period varies between 78–117 days/years.

Most individuals had established between the middle and the end of the 1990s (Fig. 7a,b). For the older classes, we observed a pronounced decrease in the number of individuals with a maximum age of 54 years (Fig. 7a,b). We observed a lower number of individuals in the lower diameter classes (less than 9 years old). In this group, we also observed that areas which remained inundated a mean of 42–78 days per year (Fig. 7a) showed an even lower number of individuals (χ^2^ = 4.17, P = P < 0.05) in the first diameter class compared to areas subjected to 79–117 days of flooding (Fig. 7b). In these last 9 years, we also observed a decrease in the annual minimum flooding levels compared to the previous 26 years (P < 0.05).

### Chronology and correlations with climatic variables

**Figure 8:**
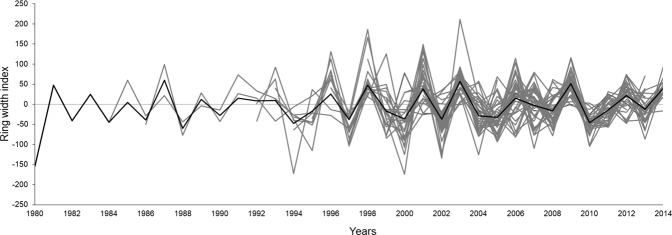
Indexed chronology of annual rings of *E. fusca* trees sampled (39 individuals) in an area of monodominance in the Pantanal subregion of Cáceres, Mato Grosso, Brazil. The gray curves represent the individual indexed curves, and the black curve represents the average chronology.

**Figure 9:**
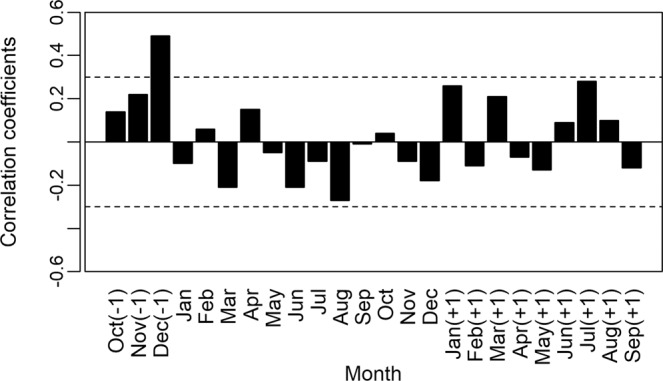
Positive correlation (December of previous year) between precipitation and *E. fusca* ring width index sampled from a monodominant stand in the Pantanal subregion of Cáceres, Mato Grosso, Brazil. Months of the growth season in the previous year are indicated with (−1) and months (+1) indicate the calendar year change during the southern hemisphere growing season (as growth begins in October). Dashed line represents significance of 0.05.

**Figure 10:**
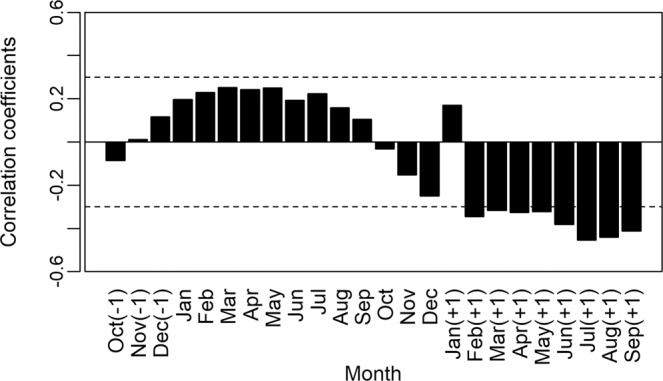
Correlations between the anomaly indices in the *El Niño* 1+2 region and the *E. fusca* ring width index sampled from a monodominant stand in the Pantanal subregion of Cáceres, Mato Grosso, Brazil. Months of the growth season in the previous year are indicated with (−1) and months (+1) indicate the calendar year change during the southern hemisphere growing season (as growth begins in October). Dashed line represents significance of 0.05.

**Figure 11:**
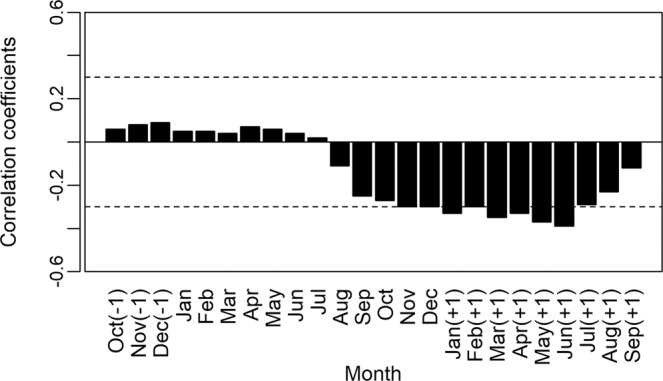
Correlations between the anomaly indices in the *El Niño* 3 region and the *E. fusca* ring width index sampled from a monodominant stand in the Pantanal subregion of Cáceres, Mato Grosso, Brazil. Months of the growth season in the previous year are indicated with (−1) and months (+1) indicate the calendar year change during the southern hemisphere growing season (as growth begins in October). Dashed line represents significance of 0.05.

The construction of an average chronology was possible for the period from 1981 to 2014 (Fig. 8), using the 36 individuals (seven destructive samples and 29 non-destructive samples). We verified a mean sensitivity of 0.477. We observed a positive correlation between the width of the rings obtained in our mean chronology and data from precipitation (December previous year) (r = −0.49, P < 0.05; Fig. 9) and a negative correlation between the width of the rings and data from the regions with *El Niño: El Niño* 1 + 2 (r = −0.38, P < 0.05; Fig. 10) and *El Niño* 3 (r = −0.34, P < 0.05; Fig. 11).

We did not observe any correlation between the width of the rings and flooding data from the study subregion (r = −0.087, P > 0.05), and we did not observe any correlation with the PDO or SOI data (r = −0.17 and r = −0.10, P > 0.05, respectively), indicating that the growth of *E. fusca* is not affected by these factors in this subregion.

## Discussion

The positive relationship between diameter and height of *E. fusca* in the study region allowed us to extrapolate an understanding of the whole structure from just the sampled population. We note a decrease in diameter increment of individuals from the seventh year onwards. The dendrochronological analyses showed that the sampled individuals of *E. fusca* (36) were young and that the oldest tree we found was 54 years. Although young, the individuals sampled were large, quite possibly the result of the rapid increase in diameter of the species, especially in the first years of life, which is an expected pattern of pioneer species^71^. The large and rapid increase in diameter in *E. fusca* has already been reported^72^.

When analyzing the age for the population as a whole (153 sampled individuals), irrespective of the period that individuals remain flooded (42–78 or 79–117 days of flooding), we observed that most of the stand was younger than 22 years, with few individuals over 40 and maximum determined age of 54 years. This low longevity of individuals probably results from the wood’s characteristics leading to breakage and rapid disintegration. This species is also characterized by low density (0.31–0.33 g cm^−3^)^73^ or (0.37 g cm^−3^)^19^ and low mechanical strength^19,20^. In the field, we observed that the trees of *E. fusca* break easily from events such as storms and that after such breakage, the stems disintegrate rapidly. The susceptibility of the species to rot when in contact with the soil was mentioned by Lorenzi^19^. In addition, we observed that the wood of the species is often attacked by wood feeder insects, which may influence its low strength and durability.

We observed a decrease in the number of individuals in first diameter class (5.41–16.97 cm) with an age of less than 9 years (Fig. 7). *E. fusca* is a monodominant, pioneer, light-demanding species^19,20^, as well as the species *V. divergens*, whose response pattern of growth rings to environmental factors are similar to our study^15,16,74^. Light-demanding species frequently have a bell-shaped distribution, with fewer individuals in larger and smaller diameter classes^75^, which shows mortality in the larger classes and low recruitment/establishment in the smaller classes, possibly related to the shading that larger individuals provide. In this work, we did not observe a well-defined bell-shape since the second and third classes presented a large number of individuals, but we observed a low recruitment/establishment in the first diameter class (Fig. 7). Whereas *E. fusca* is a light-demanding species^19,20^, this decrease in establishment over the past nine years could have been influenced by the low availability of light in the understory, possibly owing to shading of the larger individuals. Nevertheless, this pattern is not the same in different levels of inundation. We observed that this decrease match with the decline in flood levels over this period (last nine years). When we analyzed the climatic history of the region, we observed a decrease in the minimum flood level in the last nine years and, consequently, in the number of days these areas remained flooded. *E. fusca* seems to have its recruitment connected to pluriannual flood pulses, with longer periods of flooding favoring its establishment, which was apparent when we observed that areas subjected to longer flood periods (79–117 days, Fig. 7b) showed a greater number of individuals in this first diameter class when compared to areas subjected to shorter periods (between 42–78 days) (Fig. 7a). This is also supported by increased seed germination after longer periods of flooding^76^.

The mean sensitivity we observed is considered high (>0.40)^66^, which indicates high sensitivity of the species to be influenced by environmental variations. Environmental conditions greatly affect tree growth; thus, conspecific individuals can present variable growth rates, depending on the conditions to which they are exposed^23,45^. When we analyzed growth, the formation of new layers seemed to start between the months of December and January, during which time precipitation and flooding begin to increase in the Pantanal subregion. This is an unusual pattern since, for most species, wood growth seems to occur mainly during the dry season, and it is stagnant during the wet season^12,23,24^. Despite the evidence that growth starts during the period of increased flooding and precipitation, no direct statistical relationship between flooding and growth supports that idea. Although flooding influences the establishment of the species^23^, *E. fusca* growth does not seem to have a direct relationship with flooding. However, we did observe a positive correlation between precipitation in December of the previous year and *E. fusca* increment (Fig. 9). December has one of the higher precipitation levels in the Pantanal, which may indicate that the individual holds reserves during the period of greatest precipitation and then begins to grow in the next season. Fortes *et al*.^15^ also found, in Pantanal, a relationship between *V. divergens* growth and annual rainfall, but no correlation to flood patterns. In a study of two *Cedrela* species, in Brazilian Amazon, a correlation was observed between growth ring chronology and rainfall^77^.

The events of *El Niño* showed a negative impact on the growth of *E. fusca* trees. In our study, the correlation of *El Niño* 1 + 2 data with the increment of rings occurred mainly during the months of February-September (Fig. 10), while for *El Niño* 3, they were from November-June (Fig. 11). This negative correlation must be associated with the decrease in precipitation caused by *El Niño* in the Pantanal; that is, *El Niño* decreases precipitation, and, thus, the growth of *E. fusca* is negatively affected. In a study with *V. divergens* the authors also found a negative correlation between the growth and *El Niño* 1 + 2^15^. Analyses of time series of data obtained by Schöngart *et al*.^13^ indicated that during the last two centuries, the severity of *El Niño* effects has increased significantly. The events of *El Niño* significantly reduce precipitation in the Pantanal, which for *V. divergens* also resulted in a reduction in the diameter increment^15,16,74^.

Several studies conducted in tropical wetlands in Pantanal and Amazon show the positive/negative influence of *El Niño* events on tree species growth^13,15,41,73^ and in most cases *El Niño* is expected to positively affect the increment, as it increases the dry season^13,41^. Based on that, it should be recalled that our stated hypothesis held that the establishment and growth of individuals of *E. fusca* in the monodominant stands in the Pantanal subregion of Cáceres is negatively influenced by precipitation, but positively influenced by the occurrence of *El Niño*. Yet, this was refuted since the species *E. fusca* was contrary to that expected for most species. The same unusual pattern was found for *V. divergens*, which also occurs as monodominant in the northern Pantanal region^15^. This is unusual since most species are more often grow during the dry season and reduce/stagnate cambial activity during wet season due to anoxic environmental conditions^12,23,24^. The main difference between the studied area and the Amazon, were most of these studies were conducted, is the levels of inundation which is lower in Pantanal. Besides that, this species has an expressive number of adventiceous roots that can diminish anoxia effects during the wet season. This must be investigated further.

Therefore, based on the factors analyzed, we can conclude that precipitation and *El Niño* do affect the growth of the species in the study region, but that this most likely results from decreased precipitation. From the construction of a reliable chronology, we could determine that the individuals found in this monodominance of *E. fusca* in the Pantanal sub-region of Cáceres are quite young, with a maximum age of 54 years, but we could not make inferences about the age of the population, since it may have been established long before that, and individuals can be replaced. Furthermore, we observed a reduction in the number of young individuals, which appears to be a response to the decrease in the minimum flood level in the study region over the past few years. This indicates a future trend that this monodominant population will undergo a decline, if flood levels decrease, since a drastic reduction in the number of young individuals took place in less floodable areas. From these results, proposals aimed at protecting these formations can be supported, especially those preventing anthropic actions that lead to an even greater reduction in flood levels, such as dams for hydroelectric power and harbors and deforestation of the river headwaters. In addition, this study provides important information about the influence of *El Niño* on growth of arboreal species in the Pantanal, a little explored effect until now, and motivation to study this same effect with other species, including monodominant species. It also calls attention for situation of climatic changes where the levels of precipitation can diminish with changes in levels of inundation can conduct to this monodominant stand to disappear from this region.

## Data Availability

The datasets generated during and/or analysed during the current study are available from the corresponding author on reasonable request.
